# Porcine B Cell Subset Responses to Toll-like Receptor Ligands

**DOI:** 10.3389/fimmu.2017.01044

**Published:** 2017-08-25

**Authors:** Roman Othmar Braun, Sylvie Python, Artur Summerfield

**Affiliations:** ^1^Institute of Virology and Immunology, Mittelhäusern, Switzerland; ^2^Graduate School for Cellular and Biomedical Sciences, University of Bern, Bern, Switzerland; ^3^Vetsuisse Faculty, Department of Infectious Diseases and Pathobiology, University of Bern, Bern, Switzerland

**Keywords:** B cells, toll-like receptors, dendritic cells, innate immunity, vaccine adjuvants

## Abstract

Toll-like receptors (TLR) triggering of B cells are known to promote B cell expansion, differentiation of B cells into antibody-producing and memory cells, but the TLR responses of porcine B cells is poorly characterized. Therefore, this study investigated the response pattern of porcine B cell subsets to a large collection of TLR ligands and demonstrates that the TLR2 ligand Pam3Cys-SK4 and the TLR7/8 ligands gardiquimod and resiquimod are particularly efficient at inducing proliferation, CD25 and CCR7. This activation was also determined in B-cell subpopulations including a CD21^+^IgM^+^ subset, an IgG^+^ subset and two putative B1-like subsets, defined as CD21^−^IgM^high^CD11R1^+^CD11c^+^CD14^+^ and CD21^−^IgM^high^ CD11R1^−^CD11c^+^CD14^−^ B cells. The latter two were larger and expressed higher levels of CD80/86 and spontaneous phospholipase C-γ2 phosphorylation. All porcine B-cell subsets were activated by TLR2, TLR7, and TLR9 ligands. Naïve and memory conventional B cells responded similar to TLR ligands. The CD11R1^+^ B1-like subset had the highest proliferative responses. While both B1-like subsets did not spontaneously secrete IgM, they were the only subsets to produce high level of TLR-induced IgM. Similar to polyclonal IgM responses, memory B cells were efficiently induced to produce specific antibodies by CpG oligodinucleotide, resiquimod, and to a weaker extend by Pam3Cys-SK4. Depletion of plasmacytoid dendritic cells (pDCs) enhanced TLR-induced antibodies. The same set of TLR ligands also induced CD40 on cDCs, pDCs, and monocytes with the exception of TLR4 ligand being unable to activate pDCs. Gardiquimod and resiquimod were particularly efficient at inducing CCR7 on pDCs. Porcine B cells expressed high levels of TLR7, but relatively little other TLR mRNA. Nevertheless, TLR2 on B cells was rapidly upregulated following stimulation, explaining the strong responses following stimulation. Subset-specific analysis of TLR expression demonstrated a comparable expression of TLR2, TLR7, and TLR9 in all B cell subsets, but TLR3 was restricted to B1-like cells, whereas TLR4 was only expressed on conventional B cells, although both at low levels. Altogether, our data describe porcine innate B1-like cells, and how different B cell subsets are involved in innate sensing.

## Introduction

B cells are an essential component of the adaptive immune system and provide antibody-mediated protection against infections. After stimulation of surface immunoglobulin receptors and, in some cases, co-stimulatory T helper cell-derived signals, they respond with proliferation and affinity maturation of the antigen receptor, and finally differentiate into circulating memory B cells or antibody-producing plasma cells. In addition to these conventional B cells also termed B-2 cells, B-1 cells with innate functions have been described in mice and men. These cells represent the main producers of natural immunoglobulin with polyspecific reactivity against self and microbial antigens (1).

Toll-like receptors (TLR) recognize conserved pathogen-derived molecules and are central to the activation of innate immune defenses. TLR2 recognizes bacterial lipoproteins in heterodimer formation with TLR1 and TLR6 (2). TLR3 recognizes double-stranded RNA (3), while TLR4 binds to bacterial lipopolysaccharide (LPS) (4), and TLR5 to bacterial flagellin (5). TLR7/8 single-stranded viral RNA (6) and TLR9 unmethylated microbial DNA containing CpG motifs (7–9). TLR ligands are typically described as targeting phagocytes and antigen-presenting cells, which are efficient at activating innate immune responses that in turn promote adaptive immunity. In humans, B cells express TLRs 1, 6, 7, 9, and 10. Their expression and response to stimulation through a particular TLR are enhanced in memory B cells (10, 11). Murine B cells express TLR1/2, TLR4, TLR7, and TLR9 (12–14).

Although T-cell mediated B cell activation can occur in the absence of TLR signaling (15), the B cell response to antigenic stimulation is enhanced when TLRs on B cells are triggered (16, 17). In mouse models, intrinsic TLR signaling in B cells is important for IgG class-switching (18–21). This finding is of particular importance for T-independent antibody responses. Interestingly, in one study, only TLR9 ligands located within a virus-like particle triggered B cell-intrinsic TLR-enhanced antibody responses, suggesting an important role for B cell TLRs during antiviral responses (20). This finding agrees with the observation that B cell intrinsic TLR stimulation is also important for controlling persistent virus infections (22–24).

Pigs are not only an important production animal but also represent an important large animal model than can complement traditional animal models such as the mouse. Considering the important role of TLR-induced signaling in B cell responses, species-dependent differences in the response pattern, and the lack of information about this process in pigs, we investigated the response pattern of porcine peripheral B cells to a large collection of TLR ligands. We found that TLR2 and TLR7/8 ligands are particularly efficient at inducing B cell activation and proliferation, whereas TLR2, TLR7/8, and TLR9 stimulation promote antibody responses. We also analyzed the same set of TLR ligands for their ability to activate other antigen-presenting cells, altogether providing an *in vitro* evaluation of their potential as vaccine adjuvants.

## Materials and Methods

### Reagents

The TLR2 ligands Pam2Cys-Sk4, Pam3Cys-SK4, and CL429 were acquired from EMC Microcollections, Germany. The TLR3 ligand polyinosinic-polycytidylic acid (poly I:C) was purchased from Sigma-Aldrich, Switzerland. The TLR4 ligands Kdo2-Lipid A, monophosphoryl lipid A (MPLA), and lipid A detoxified were purchased from Avanti Polar Lipids, USA. The TLR4 ligand LPS (*E. coli*-derived) was acquired from Sigma-Aldrich. The TLR5 ligand Flagellin (FLA-ST, *S. typhimurium*-derived) was purchased from Invivogen, France. The TLR7 ligands loxoribine and gardiquimod, as well as the TLR7/8 ligand resiquimod were acquired from Chemdea (Ridgewood, NJ, USA), and the TLR7 ligand imiquimod was purchased from Invivogen. The TLR9 ligands CpG oligodinucleotide (ODN) D32 (5′-ggT GCG TCG ACG CAG ggg gg-3′), CpG 2007 (5′- tcg tcg ttg tcg ttt tgt cgt t -3′), and CpG 2142 (5′-TCGCGTGCGTTTTGTCGTTTTGACGTT-3′) were ordered from Eurofins, Switzerland. ODN D-SL01 (5′-tcgcgacgttcgcccgacgttcggta-3′) and ODN D-SL03 (5′-tcgcgaacgttcgccgcgttcgaacgcgg-3′) were purchased from Invivogen or Eurofins, Switzerland. The positive control for proliferation concanavalin A (ConA) was acquired from Sigma-Aldrich.

CellTrace Violet stain was purchased from Thermofisher Scientific.

Monoclonal antibodies used included CD4 (clone 74-12-4; ATCC), CD14 (clone MIL2; Biorad, USA), CD16 (clone G7, Biorad), CD21 (clone BB6-11C9.6; Southern Biotech, USA), CD40 (clone G28.5, ATCC, USA), CD79a-APC (clone HM57, BioLegend, USA), CD80/86 (detection through human cytotoxic T-lymphocyte associated antigen-mouse immunoglobulin fusion protein; Enzo Biochem, USA), CD115 (clone ROS8G11-1; kindly provided by Prof. David Hume, Roslin Institute, University of Edinburgh, UK), CD172a (clone 74-22-15A, hybridoma kindly provided by Prof. Armin Saalmüller, Veterinary University, Vienna, Austria), CD172a-PE (Southern Biotech, USA), anti-IgM (clone PIG45A, ATCC), anti-IgG (23.7.1b, Thermofisher Scientific, Switzerland), anti-CD25 (clone K231.3B3, Biorad), and CADM1 (clone 3E1, MBL International, USA). Anti-TLR2 antibodies were kindly provided by Dr. Javier Dominguez (Departemento de Biotecnología, Instituto Nacional de Investigación y Tecnología Agraria y Alimentaria, INIA, Madrid, Spain). Recombinant porcine IL-3 his-tagged (IL-3-his) was produced as described (25). Anti-his-tag antibody was purchased from Roche, Switzerland. Phosphorylated phospholipase (PLC)-γ2 was detected using Alexa Fluor^®^ 647 mouse anti-PLC-γ2 (pY759) (clone K86-689.37, Becton Dickinson). Anti-mouse secondary antibodies were purchased from Invitrogen (AF488, AF647, and PE-Cy5.5), Southern Biotech (FITC and PE), Abcam (PE-Cy7), and BD (V500 and V421 conjugates).

### Cells

Citrated blood was taken from a total of eight different pigs housed in our specific pathogen-free facility. Blood withdrawal was performed under the guidance of the Swiss animal welfare law, approved by the Cantonal ethical committee for animal experiments (BE88/14 and BE90/14). Peripheral blood mononuclear cells (PBMCs) were prepared using density gradient centrifugation (1,000 × *g*, 25 min) over Ficoll-Paque (1.077 g/L, GE Healthcare Life Science). CD21^+^ B cells were isolated using magnetic cells sorting (MACS), following the manufacturer’s instructions (Miltenyi Biotec, Germany). PBMCs were cultured in DMEM + GlutaMAX (Thermofisher, Switzerland) supplemented with 10% fetal bovine serum (Thermofisher) and 0.02 mM beta-mercaptoethanol (Sigma-Aldrich) at 39°C and 5% CO_2_. CD21-sorted B cells were cultured in medium and conditions as described above, supplemented with HEPES, non-essential amino acids, sodium pyruvate (Thermofisher), and 50 U/ml recombinant porcine interleukin-2 (IL-2, kindly provided by Dr. S. Inumaru, National Institute of Animal Health, Ibaraki, Japan).

Peripheral blood mononuclear cells lacking plasmacytoid dendritic cells (pDCs) were obtained by depleting IL-3 receptor expressing cells as described (26). Briefly, PBMCs were incubated with porcine IL-3-his followed by anti-His-IgG (Roche), anti-IgG-microbeads (Miltenyi Biotec). All incubations lasted for 15 min on ice. After washing, cells were passed through an LD column and the negative fraction was collected on ice.

Purified B-cell subsets were obtained using FACS sorting. After PBMC preparation, T cells were depleted using the MACS system (Miltenyi Biotec). After incubation with anti-CD3 (clone PTT3/FyH2), cells were incubated with anti-mouse IgG magnetic beads (Miltenyi Biotec) and sorted using an LD column. The CD3^−^ fraction was then stained with anti-CD21, IgM, IgG, and CD11R1 as described in the Section “Results”. After doublet exclusion and lymphocyte gating, CD21^+^IgM^+^ (P1), IgG^+^ (P2), CD21^−^IgM^+^CD11R1^+^ (P3), and CD21^−^IgM^+^CD11R1^−^ (P4) cell subsets were defined by electronic gating and the four subsets sorted using a fluorescence activated cell sorting (FACS) Aria (BD Biosciences).

### Detection of B-Cell Activation Markers

Peripheral blood mononuclear cells were seeded at 1 × 10^6^ cells/500 μl and stimulated with TLR ligands for 17 h. Then, cells were harvested and stained for B cell subsets using anti-IgM, -IgG, CD21, and CD11R1, combined with CD25 or CCR7 as activation markers.

To quantify tonic B cell signaling, freshly isolated PBMC were first stained for surface markers, incubated for 2 min, then fixed and permeabilized (BD Phosphoflow^TM^ reagents) and stained for PLC-γ2 (pY759).

### Proliferation Assays

Peripheral blood mononuclear cells were cultured in medium as described above at a concentration of 2 × 10^6^ PBMCs/ml in round-bottom microtiter plates They were stimulated with TLR ligands at the following concentrations: 10 µg/ml for TLR2 ligands, 1 µg/ml for TLR4 ligands, 1 µg/ml for TLR5 ligands, 5 µg/ml for TLR7/8 ligands, and 5 µg/ml for TLR9 ligands. The cells were incubated for 2 days, and 1 μCi ^3^H-thymidine per well was added during the final 18–24 h. Cells were harvested on glass fiber filters and radioactivity was quantified in a Microbeta 1640 Trilux counter (Perkin Elmer).

To identify proliferating cell populations, we used CellTrace Violet stain and multicolor flow cytometry. Fifty million PBMCs or enriched B cells were resuspended in 1 ml PBS. CellTrace Violet was added followed by incubation at 37°C for 10 min. Next, 2 ml FBS was added to the suspension and it incubated at room temperature in the dark for 15 min. Cells were washed twice with PBS/EDTA and centrifuged at 350 × *g* at room temperature for 10 min. Cells were then seeded into round-bottom 96-well plates at 200,000 cells/well in 200 µl final volume, with TLR ligands at the concentrations described above. After incubation at 39°C/5% CO_2_ for 5 days, cells were stained with primary and secondary antibodies for B cell subsets corresponding to the desired read-out. IgG block (Jackson Immunoresearch, USA) was performed before adding primary antibodies when using enriched B cells.

### *In Vitro* Total IgM Production

Peripheral blood mononuclear cells or purified B cell subsets were cultured for 5–7 days culture at 39°C/5% CO_2_ at the conditions indicated in the figure legends, and supernatants were harvested and frozen until analysis. In some cultures, 50 U/ml recombinant porcine IL-2 (kindly provided by Dr. S. Inumaru, National Institute of Animal Health, Ibaraki, Japan) and 10 ng/ml recombinant porcine B-cell activating factor [BAFF, prepared as previously described (27)] were added. Nunc-Immuno 96-well plates (Sigma-Aldrich) were coated with anti-IgM antibody in PBS (clone 5C9, 1:200). After overnight incubation at room temperature, plates were washed three times with wash buffer (PBS + 0.05% Tween 20) and blocked with PBS/0.5% BSA/0.05% Tween 20 buffer at 37°C for 1 h. After washing, samples were transferred and plates incubated at 37°C for 2 h. Next, plates were washed three times and we added goat anti-pig detection antibody coupled with horseradish peroxidase (Bethyl, A100-117P, 1:20,000) for 20 min at 37°C. After washing, the substrate OPD (Sigma-Aldrich) was added and absorbance was measured at 450 nm using VersaMax reader (Molecular Devices, USA).

### Memory B Cell Restimulation

Two pigs were vaccinated with a commercial vaccine against FMDV A Iran 96 (kindly provided by Merial, Pirbright, UK) using a prime boost vaccination protocol with 4 weeks between injections. PBMCs from these animals were used 3–7 months after booster vaccination. Cells were cultured in 24-well plates at a concentration of 2 × 10^6^ cells/well and stimulated with purified FMDV antigen (10 µg/ml 146S antigen derived from A Iran 96, kindly provided by Merial) and/or TLR ligands, and incubated for 7 days at 39°C, 5% CO_2_.

FMDV-specific antibodies were detected by ELISA. Plates were coated with 100 µl 1 µg/ml FMDV A Iran 146 S antigen in PBS and incubated over night at 4°C. After washing with PBS, the plates were blocked with 1% BSA in PBS for 1 h at room temperature. Then, samples were applied and incubated for 30 min at room temperature. After washing the plates with PBS, peroxidase-conjugated goat anti-swine IgG (Jackson ImmunoResearch, PA, USA) followed by the addition of TMB as substrate.

### Reverse Transcription-Polymerase Chain Reaction (RT-PCR) for TLR Expression

B cells and monocytes were enriched with MACS using CD21 and CD14 antibodies, respectively. Purified pDCs were obtained using fluorescence activated cell sorting (FACSAria, Becton Dickinson) based on their CD172a^+^CD4^high^ phenotype (28). The purity of sorted cells was over 98%. The purified B cell subsets P1–P4 were also obtained by FACS sorting as described above. After sorting, cells were washed and resuspended in TriZOL for RNA extraction using an RNeasy mini kit (Qiagen, Switzerland). RNA was treated with RNase-free DNase I (Ambion, UK). RT-PCR was performed on a Thermocycler ABI 7500 (ThermoFischer). RNA expression levels were calculated based on 18S RNA levels. TLR2 forward primer, 5′-TATCCAGCACGAGAATACACAGTTTAA-3′; TLR2 reverse primer, 5′-CGAGTTGAGATTGTTATTGCTAATATCTAAAA-3′; TLR2 probe, 5′-CATTGGCTTCCCCAGACCCTGGA-3′; TLR3 forward primer, 5′-AAAATCTCCAAGAGCTTCTATTAGCAA-3′; TLR3 reverse primer, 5′-TTGTATTTGATTTGATGACAACTCTAATCTTT-3′; TLR3 probe, 5′-CGTGAAGAACTTGATTTCCTTGGCAATTCTTC-3′; TLR4 forward primer, 5′-TGGCAGTTTCTGAGGAGTCATG-3′; TLR4 reverse primer, 5′-CCGCAGCAGGGACTTCTC-3′; TLR4 probe, 5′-CGGCATCATCTTCATCGTCCTGCAG-3′; TLR7 forward primer, 5′-CAGAAGTCCAAGTTTTTCCAGCTT-3′; TLR7 reverse primer, 5′-GGTGAGCCTGTGGATTTGTTG-3′; TLR7 probe, 5′-CAACTGGTACATCAGCACCTCTCAAGCAGA-3′; TLR8 forward primer, 5′-GATACCATTGCGGCGATAATATG-3′; TLR8 reverse primer, 5′-TTTACCTTGGCTAAGCACACATG-3′; TLR8 probe, 5′-ATGTTGGCTGCCCTGGCTCACC-3′; TLR9 forward primer, 5′-GAGACCCTGCTGTTGTCCTACAA-3′; TLR9 reverse primer, 3′-AGTTCCCCCCCACATCAA-5′; TLR9 probe, 5′-CGCCTGAGGACCTGGCCAATGTG-3′.

### Dendritic Cell Subset Activation

Peripheral blood mononuclear cells were cultured in 12-well plates at 2 × 10^6^ cells/ml in 2 ml. Cells were stimulated with TLR ligands and incubated for 5 h at 39°C/5% CO_2_. Next, cells were harvested and stained with markers for CD4, CD14, CD80/86, CD172a, CCR7, and CADM-1. Samples were read using a FACSCanto II and data were analyzed with FlowJo software. Monocytes were identified and gated as CD14 cells, conventional DC1 (cDC1) as CD14^−^CD4^−^CADM1^+^CD172^low/−^ cells, cDC2 as CD14^−^CD4^−^CADM1^+^CD172^+^ cells, and pDC as CD14^−^CD4^+^CADM1^−^CD172^low/+^ cells (26, 29).

### Statistical Analysis

Analysis was performed in Prism 7 (GraphPad, USA). All statistical analyses used parametric tests for paired samples. ANOVA with Greenhouse-Geisser correction followed by Tukey’s test was performed for multiple comparisons of the mean of each group with the control, and Dunnett’s test was used generate *P* values for each comparison. Where applicable two-way ANOVAs were performed followed by Sidak’s multiple comparison tests. *P*-values were defined as follows: ****P < 0.0001; ***P < 0.001; **P < 0.01; *P < 0.05; ns: not significant. Correlation was calculated using the linear regression method.

## Results

### Strong B Cell Proliferative Response after PBMC Stimulation with TLR2, 7/8, and 9 Ligands

**Figure 1:**
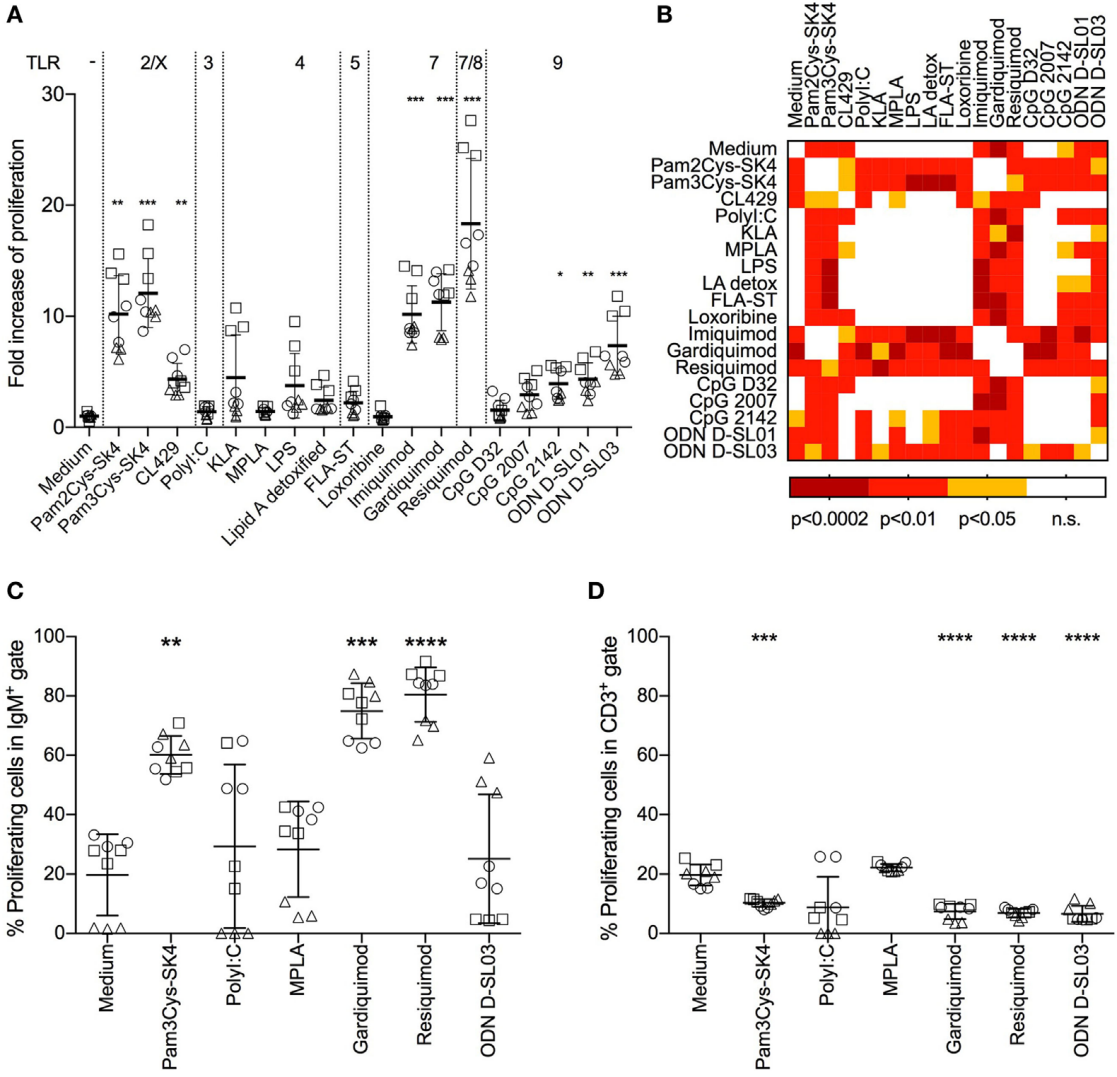
Proliferative response of B cells in whole peripheral blood mononuclear cells (PBMCs) after stimulation with toll-like receptor ligands. **(A)** Proliferation of PBMCs after stimulation for 3 days was determined using a ^3^H-thymidine assay. The fold increase over the medium control is shown. **(B)** Heatmap giving an overview of the statistical significances found between the treatments shown in **(A)**. **(C)** Proliferation of IgM^+^ B cells determined by flow cytometry using CellTraceViolet. The percentage of proliferating cells in the IgM gate is shown. **(D)** Proliferating CD3^+^ T cells, again determined using CellTrace Violet. The percentage of proliferating cells in the CD3 gate is shown. **(A,C,D)** PBMC culture triplicates from three different animals, each depicted by a different shape. Mean values are indicated by horizontal bars. Error bars show SDs. Statistical significance was calculated using ANOVA followed by Tukey’s test (****P < 0.0001; ***P < 0.001; **P < 0.01; *P < 0.05; n.s., not significant).

Stimulating TLRs in immune cells engages various pathways, resulting in surface marker upregulation, cytokine production, and cell proliferation. As a first step, we tested the stimulatory potential of TLR ligands by assessing their ability to induce proliferation of whole PBMCs. We screened a set of 18 TLR ligands using a ^3^H-thymidine incorporation assay. We tested ligands described as stimulating TLR2/1 and TLR2/6 heterodimers, TLR3, TLR4, TLR5, TLR7, TLR8, and TLR9 (Figure 1A).

Pam3Cys-SK4, a TLR2/1 ligand, and Pam2Cys-SK4, a TLR2/6 ligand, induced proliferation 8–18-fold over control values. CL429 (a conjugate of a TLR2/1 ligand with a NOD ligand) induced lower levels of activation (2–7-fold over controls). No significant difference between Pam3Cys-SK4 and Pam2Cys-SK4 was detected (Figure 1B). The TLR3 ligand Poly I:C, all TLR4 ligands and the TLR5 ligands flagellin induced no significant proliferation compared to controls. Among the TLR7 ligands, imiquimod and gardiquimod induced potent proliferative responses in the range of those induced by Pam3Cys-SK4, while loxoribine was not active. Resiquimod, a TLR7/8 ligand, induced the highest proliferation of all ligands tested, with values 11–27-fold those of controls. For stimulation of TLR9, various CpGs were tested. The type A CpG D32 and the type B CpG2007 did not induce significant responses. CpG2142 induced a proliferation up to 5-fold over controls. Type B ODNs D-SL01 and type C D-SL03 induced the highest proliferation of this group, with proliferation values 7 and 12 times those of controls, respectively.

We identified B cells as the main proliferating population after TLR ligand stimulation. To this end, we employed multicolor staining for B cells (IgM^+^) and T cells (CD3^+^), with CellTrace Violet stain as a proliferation marker. Interestingly, up to 80% of IgM^+^ cells proliferated when stimulated with the TLR7/8 ligand resiquimod (Figure 1C), whereas no response or even suppression was found in T cells with the selected TLR ligands (Figure 1D).

### Porcine B Cell Subsets Definition

**Figure 2:**
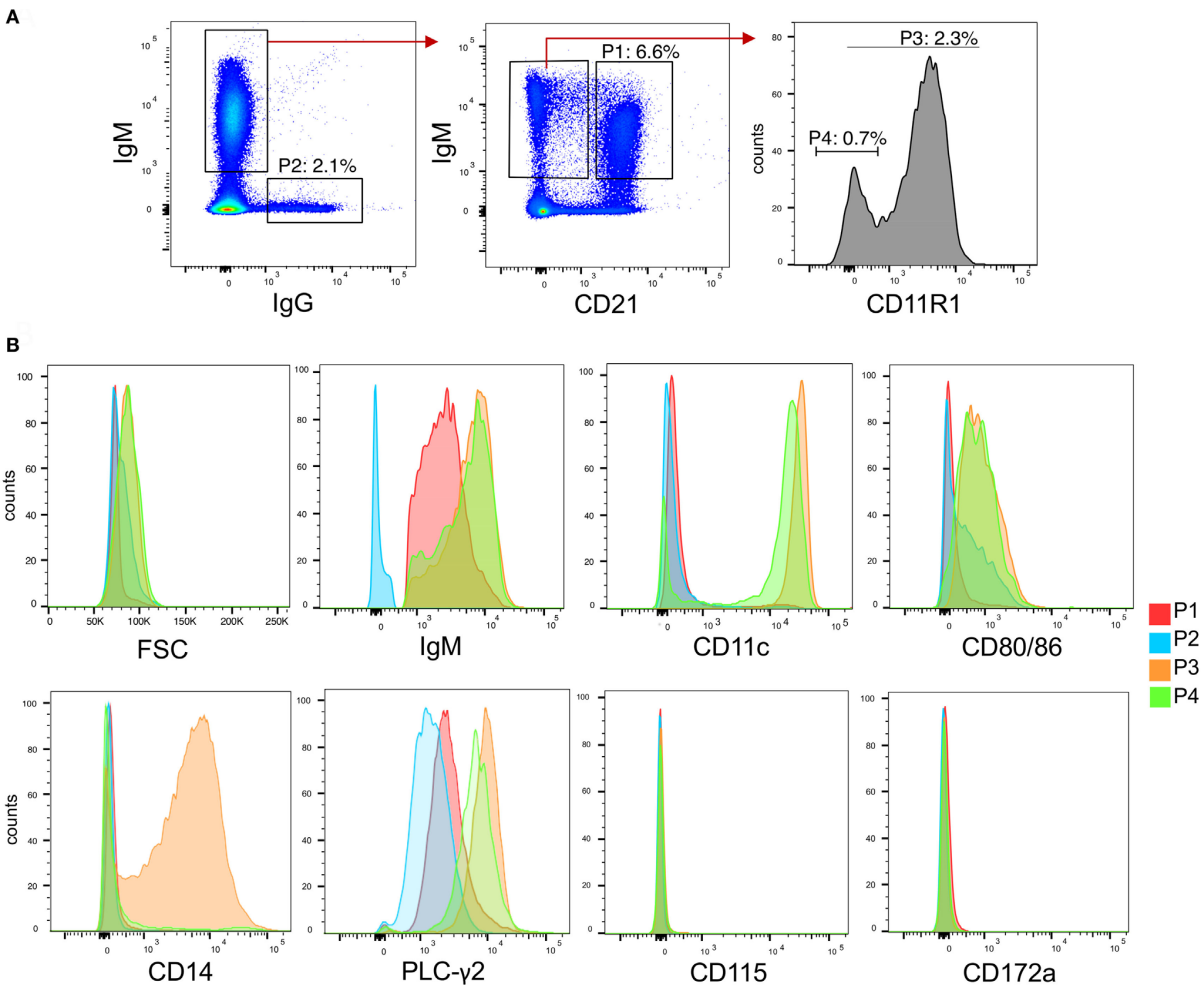
B cell subsets in peripheral blood of pigs. **(A)** Gating strategies for identifying B cell subsets based on expression of IgG, IgM, CD21, and CD11R1. After doublet exclusion, population 1 (P1) was defined as CD21^+^IgM^+^, P2 as IgG^+^, P3 as CD21^−^IgM^+^CD11R1^+^, and P4 as CD21^−^IgM^+^CD11R1^−^. **(B)** Histogram overlays of FSC, IgM, CD11c, CD80/86, CD14, phosphor-PLC-γ2, CD115, and CD172a for the subsets P1–P4 identified in **(A)**. A representative animal out of three is shown.

To identify different cell subsets in peripheral blood, we established multicolor staining for surface IgM, IgG, CD21, and CD11R1 expression. The CD11R1 molecule in pigs is recognized by cross-reactive CD11b antibodies but differ in its cellular distribution (30). After excluding doublets, we identified four B cell populations (Figure 2A). P1 was defined as IgM^+^CD21^+^ cells and is composed of mainly naïve B cells (31). P2 represented IgG-switched memory B cells. The two novel B-cell subsets P3 and P4 were defined as CD21^−^IgM^+^CD11R1^+^ and CD21^−^IgM^+^CD11R1^−^, respectively.

To further characterize P3 and P4, we analyzed various parameters by flow cytometry (Figure 2B). Forward-scatter analysis showed that the two IgM^+^CD21^−^ subsets were bigger compared P1 and P2. Both P3 and P4 also expressed higher levels of IgM when compared to P1. CD11c was shown to be expressed at high level on P3 and on a major part of P4 but not on P2. P3 and P4 were also found to express higher levels of CD80/86, when compared to P1 and P2. Only P3 expressed CD14.

The higher size and surface IgM levels is a characteristic of B-1 cells (1) indicating that both P3 and P4 could represent the porcine equivalent of B-1 cells. Furthermore, as shown in Figure 2B, the phenotype of P3 was reminiscent to that of a subset of human B-1 cells expressing high levels of CD11b, CD11c, CD14, and CD86 (32, 33). We therefore also tested if P3 and P4 had a higher level of tonic intracellular signaling, a characteristic of B-1 cells (32, 34) by analyzing their expression of phosphorylated PLC-γ2, and found this to be elevated when compared to P1 and P2 subsets (Figure 2B).

Contrasting with monocytes and blood DC (29), none of the B-cell subsets expressed CD115 and CD172a. We therefore propose that P3 and P4 represent innate B cell subsets with B-1 like functions.

### Activation Marker Response of B Cells to TLR Stimulation

**Figure 3:**
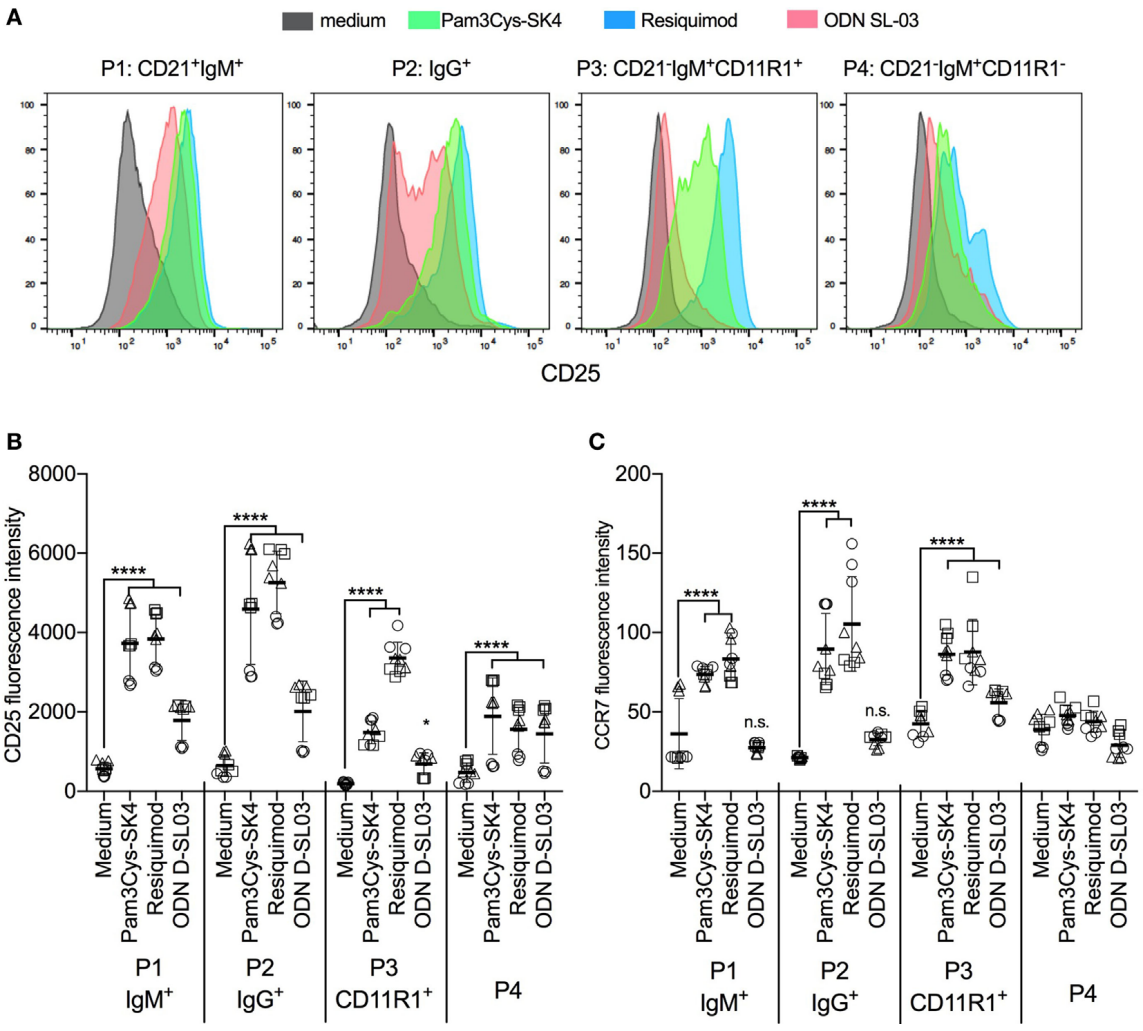
Activation markers on B cell subsets after stimulation with toll-like receptor (TLR) ligands. **(A)** Histogram overlays of CD25 expression on the B cell subsets P1–P4 defined as shown in Figure 2. Peripheral blood mononuclear cells (PBMCs) were cultured and stimulated overnight and B-cell subset identified using multicolor flow cytometry. A representative animal out of three is shown. **(B,C)** Fluorescence intensity of CD25 and CCR7 after stimulation of PBMC with TLR ligands. PBMCs were stimulated overnight and B-cell subsets identified using multicolor flow cytometry. Triplicates cultures from three different animals, each depicted by a different symbol, are shown. Mean values are indicated by horizontal bars. Error bars show SDs. Statistical significance was calculated using ANOVA followed by Tukey’s test.

To evaluate the responses of the B-cell subsets P1–P4 to TLR stimulation, PBMCs were stimulated with the most potent TLR ligands identified in the screening experiments shown in Figure 1. Pam3Cys-SK4 and resiquimod induced strong upregulation of CD25 in all subsets (Figures 3A,B), while responses to ODN D-SL03 induced upregulation in all subsets with the exception of P3. This population was also less responsive to Pam3Cys-SK4 when compared to resiquimod. We also compared the relative responsiveness of the B-cell subsets by calculating the fold-increase in CD25 expression induced by TLR stimulation. While the response of P1 and P2 were comparable (6–7-fold increase of CD25 following resiquimod stimulation), P3 was found to be particularly responsive to resiquimod (17-fold increase). P4 was the subset with the overall lowest TLR responsiveness (3.3-fold increase).

Also, when analyzing CCR7 we found the strongest induction by Pam3Cys-SK4 and resiquimod (Figure 3C). In this assay, P1 and P2 were unresponsive to ODN D-SL03. Furthermore, no upregulation of CCR7 was detected in P4 after stimulation.

### CD11R1^+^ B-1 Like Porcine B-Cells Have the Highest Proliferative Capacity in Response to TLR Stimulation

**Figure 4:**
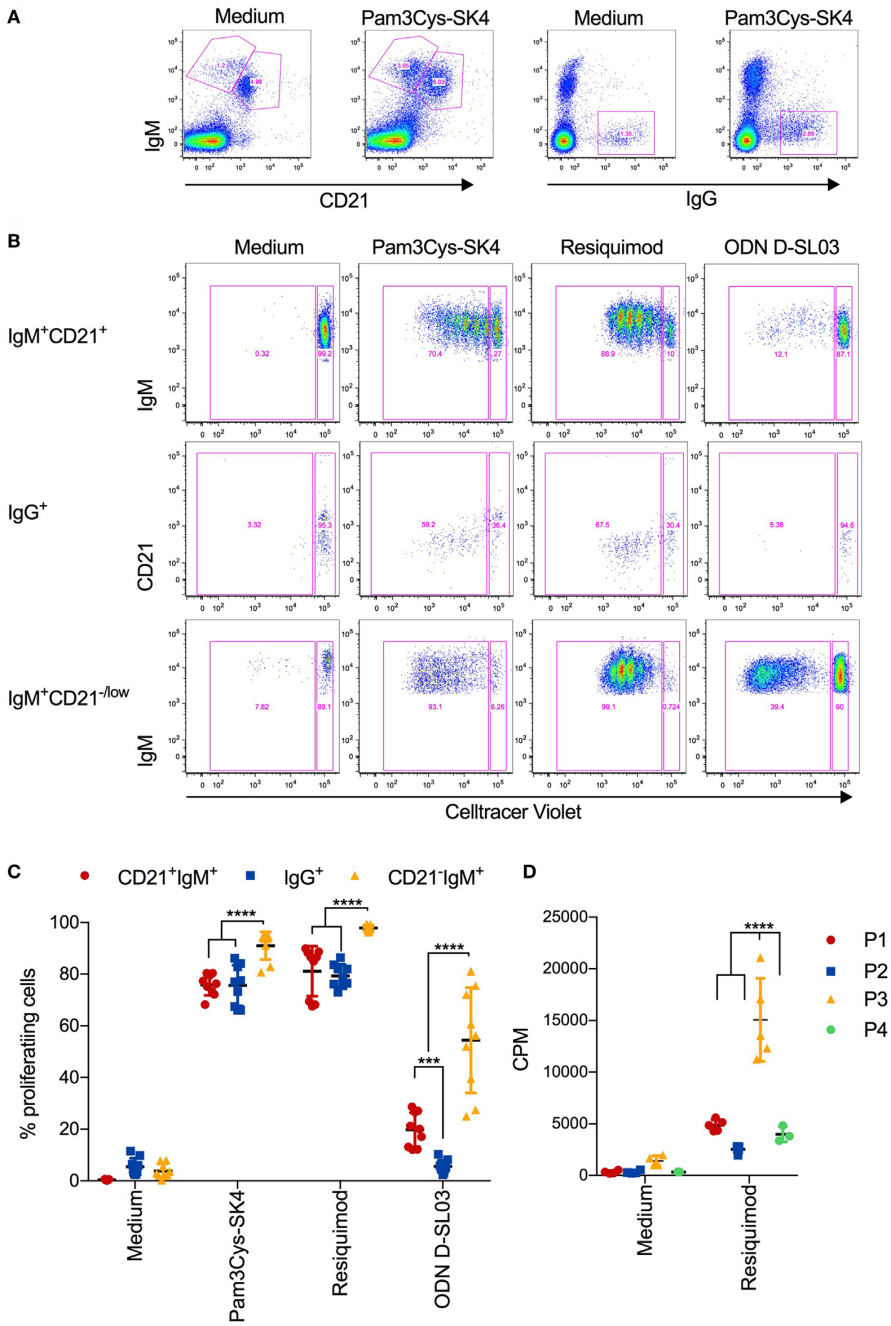
Proliferative responses of B cell subsets after stimulation with toll-like receptor (TLR) ligands **(A)** Gating strategy defining CD21^+^IgM^+^, CD21^+^IgM^+^, and IgG^+^ B cells, exemplified for unstimulated and Pam3Cys-SK4 stimulated peripheral blood mononuclear cells (PBMCs) after 5 days of culture. **(B)** CellTrace Violet staining of the three B-cell subsets defined in **(A)** using multicolor flow cytometry. PBMCs were cultured for 5d. A representative animal out of three is shown. **(C)** Percentage of proliferating B cell subsets, defined as shown in **(A,B)**, after stimulation of PBMCs with the indicated TLR ligands. Triplicates cultures from three different animals are shown. **(D)** Proliferation of FACS-sorted B cell subsets after stimulation for 3 days was determined using ^3^H-thymidine incorporation. P1 was defined and sorted as CD21^+^IgM^+^ subset, P2 as IgG^+^ subset, P3 as CD21^−^IgM^+^CD11R1^+^ subset, and P4 as CD21^−^IgM^+^CD11R1^−^ subset (for gate definition see Figure 2). **(C,D)** Mean values are indicated by horizontal bars. Error bars show SDs. Statistical significance was calculated using ANOVA followed by Tukey’s test.

We next investigated the proliferative response of B cell subsets using multicolor flow cytometry with CellTrace Violet staining. The identification of P1-P4 B-cell subsets was not possible due to an apparent loss of CD11R1 expression in culture after 5 days (data not shown). We therefore gated for CD21^+^IgM^+^, CD21^−/low^IgM^+^, and IgG^+^ cells subset (Figure 4A). A representative CellTrace Violet staining of the three subsets is shown in Figure 4B and a summary of all biological replicates in Figure 4C. Similar to the induction of CD25 and CCR7 expression, Pam3Cys-SK4 and resiquimod were found to be the most potent inducers of proliferation. Only the IgG^+^ B-cell subset was unresponsive to the CpG stimulation. When comparing the three gated subsets, we found the CD21^−/low^IgM^+^ subset to have the strongest response to all three TLR ligands.

Considering the uncertainty with respect to the identity of the above B-cell subsets, we FACS-sorted the four B-cell subsets P1–P4 before stimulation with resiquimod and determined the proliferation by ^3^H-thymidine incorporations as in Figure 1A. The results demonstrated that P3 was the B-cell subset with the highest proliferative potential (Figure 4D).

### CD21^−^IgM^+^ B Cell Subsets Are the Main Source of IgM Following TLR Stimulation

**Figure 5:**
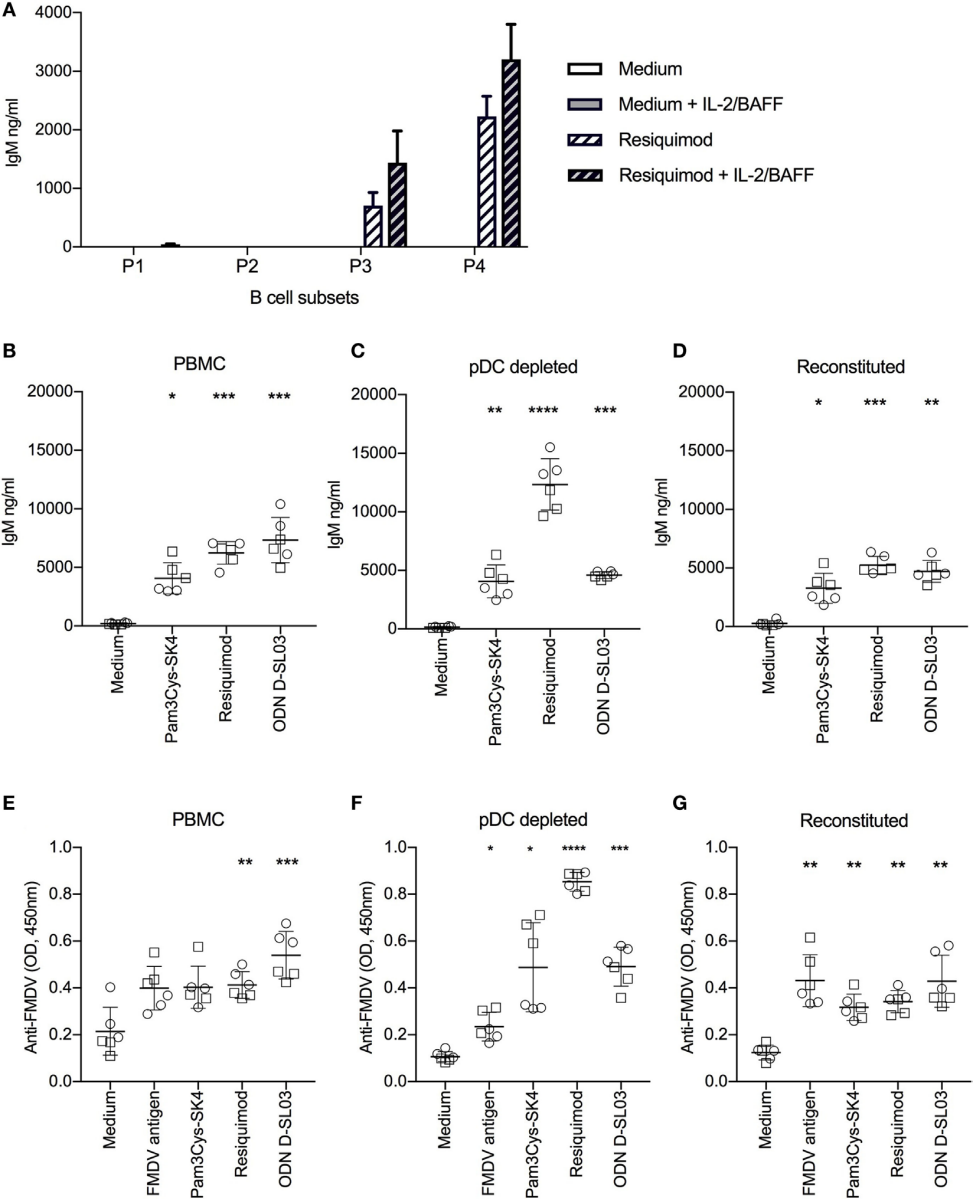
Antibody production after stimulation with toll-like receptor (TLR) ligands. **(A)** Spontaneous and induced IgM responses by B cell subsets. The B cell subsets P1–P4 defined as shown in Figure 2 were FACS-sorted, cultured at 0.5 × 10^6^ cells/ml and stimulated with resiquimod in the absence or presence of interleukin-2 (IL-2) and B-cell activating factor (BAFF) for 5 days. IgM production in the supernatants was quantified by ELISA. **(B)** TLR-induced IgM production induced of full PBMCs, of plasmacytoid dendritic cell (pDC)-depleted PBMCs **(C)**, and of PBMCs reconstituted with pDC following sorting **(D)**. **(E–G)** FMDV-specific IgG-antibody levels detected in the same setup as in **(B–D)**. **(B–G)** Cells were cultured for 7 days. Six biological replicates of two independent experiments are shown. Statistical significance was calculated using ANOVA followed by Tukey’s test (****P < 0.0001; ***P < 0.001; **P < 0.01; *P < 0.05).

Considering that spontaneous IgM secretion has been found as a characteristic of murine and human B-1 cells (33, 35), we tested this function in sorted porcine B cell subsets P1–P4. As shown in Figure 5A, high levels of IgM were only found in the supernatants of TLR-stimulated P3 and P4 B-cell subsets. Even the addition of recombinant porcine IL-2 and BAFF, both previously shown to be required for *in vitro* activation of porcine memory B cells to differentiate into antibody-producing cells (36), did not permit spontaneous IgM secretion by any B-cell subset. Nevertheless, in the presence of the cytokines the CD21^+^IgM^+^ P1 subset produced 38 ng/ml, but this value was still 39 and 85 times lower that the production found with P3 and P4, respectively. Altogether, this support that P3 and P4, although apparently unable to constitutively produce IgM, possess B1-like characteristics not found with conventional B cells.

### TLR Ligands Promote Polyclonal IgM and Memory B Cell Differentiation to Antibody-Producing Cells

Next, we compared polyclonal IgM secretion and restimulation of FMDV-specific memory B cells to produce antibodies following TLR stimulation and found potent responses with Pam3Cys-SK4, resiquimod and ODN D-SL03 (Figures 5B,E). Considering that pDCs play an important role in human B cell activation *in vitro* (37), we tested the effect of pDC depletion on antibody responses. Also in absence of pDC, B cells were able to produce IgM and virus-specific antibodies following TLR stimulation. Interestingly, antibody levels were significantly increased following resiquimod stimulation but not with the other TLR ligands (Figures 5C,F). When the sorted ILR3^+^ fraction containing the pDC was pooled with the negative fraction (“reconstituted”), the values obtained were again comparable to the PBMC controls indicating that the higher levels of IgM following resiquimod stimulation was not an effect of the sorting process (Figures 5D,G). In contrast to the promoting effect of pDC depletion on resiquimod stimulation, it resulted in reduced responses to FMDV antigen (PBMC compared to pDC-depleted *P* = 0.019 and pDC-depleted compared to reconstituted *P* = 0.01).

### TLR Expression Levels in Porcine B Cells

**Figure 6:**
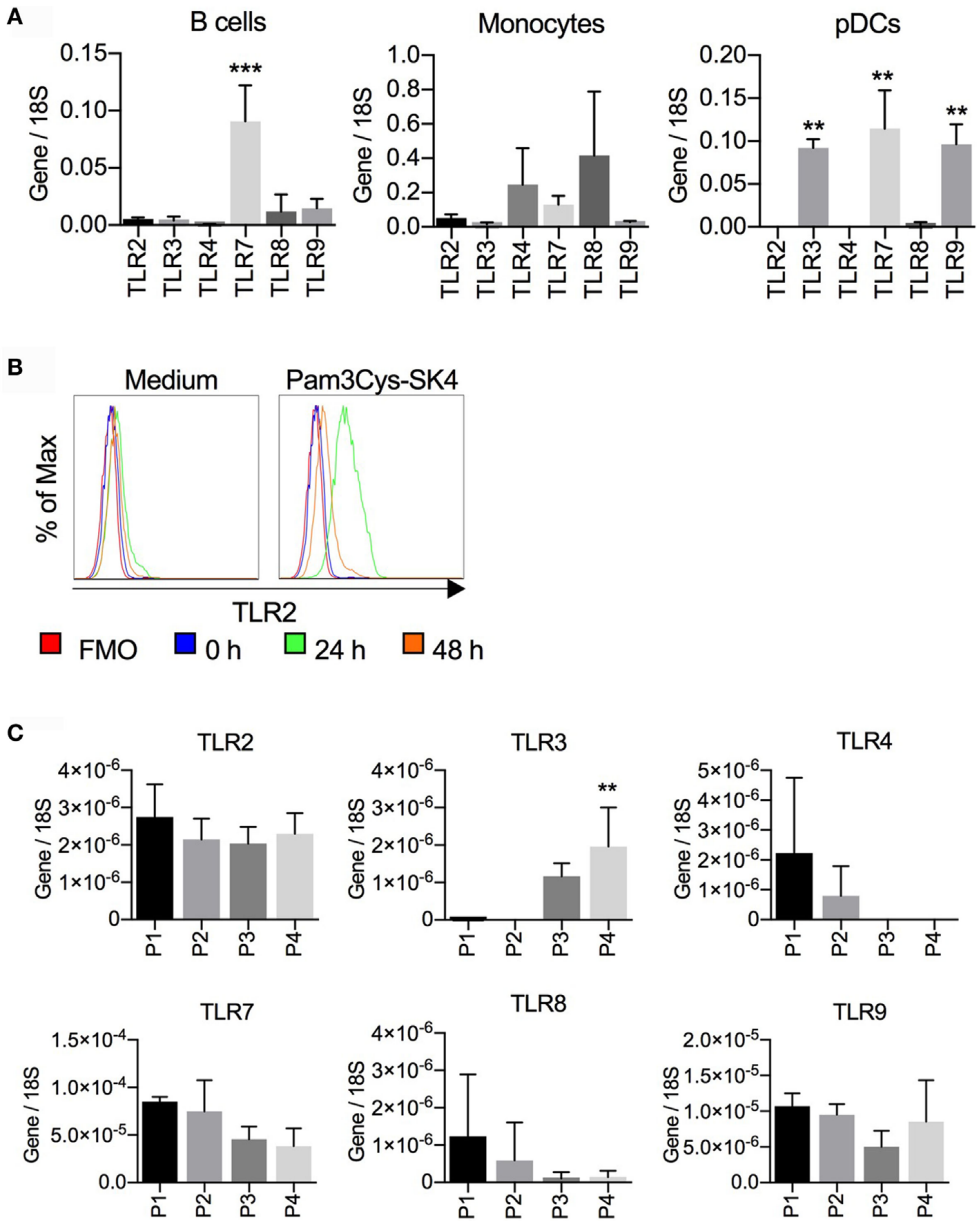
Toll-like receptor (TLR) mRNA expression in B cells, monocytes, and plasmacytoid dendritic cells (pDCs). **(A)** CD21^+^ B cells and CD14^+^ monocytes were sorted by magnetic cells sorting (purity > 98%); CD172a^+^CD4^high^ pDCs were purified by fluorescence activated cell sorting (FACS) (purity > 99%). RNA was isolated for RTqPCR analysis of TLR expression. Data are from three biological replicates generated using cells from three different pigs. In B cells, *TLR7* expression was significantly higher than all other TLR (***P < 0.0001). In pDC, TLR3, TLR7, and TLR9 were found to be significantly higher when compared to the other TLRs (**P < 0.001). **(B)** Expression of TLR2 on CD21^+^ cells after stimulation of peripheral blood mononuclear cells with Pam3Cys-SK4 for 24 h. A representative experiment from three independent experiments is shown. **(C)** TLR mRNA expression of B cell subsets P1–P4 sorted by FACS according to the gates defined in Figure 2. Mean values calculated from three different animals with standard deviations are shown. P4 was statistically significant from P1 and P2 for TLR3 expression (*P < 0.05).

To better understand B cell response patterns to TLR ligand stimulation, we investigated TLR expression at the transcriptional level using RT-qPCR, and compared results to other antigen-presenting cells. We first compared MACS-sorted CD21^+^ B cells, CD14^+^ monocytes, and FACS-sorted CD172a^+^CD4^+^ pDCs (Figure 6A). B cells expressed high levels of *TLR7*, intermediate levels of *TLR8* and *TLR9*, and low but detectable levels of *TLR2, TLR3*, and *TLR4*. Dominant TLR genes on monocytes were *TLR4* and *TLR8* (although statistically not significant different from other TLRs) and on pDC *TLR3, TLR7*, and *TLR9*.

Considering the strong responses of B cells to TLR2 ligands, we tested if TLR2 was upregulated following stimulation, as has been reported for human B cells. PBMCs were stimulated with Pam3Cys-SK4 and surface expression of TLR2 on CD21^+^ cells was analyzed by flow cytometry. After 24 h, a potent upregulation of TLR2 was detected (Figure 6B).

We next analyzed TLR expression by RT-qPCR on sorted B-cell subsets as defined in Figure 2. Only the expression of TLR3 on the P4 subset was found to be significantly higher when compared to P1 and P2. Nevertheless, TLR3 transcripts were also found in P3 but were absent in P1 and P2. This contrasted with the TLR4 expression, which was undetectable in the B-1 like subsets but present in P1 and P2, although at low levels (Figure 6C).

### Activation Marker Response of DC Subsets and Monocytes

**Figure 7:**
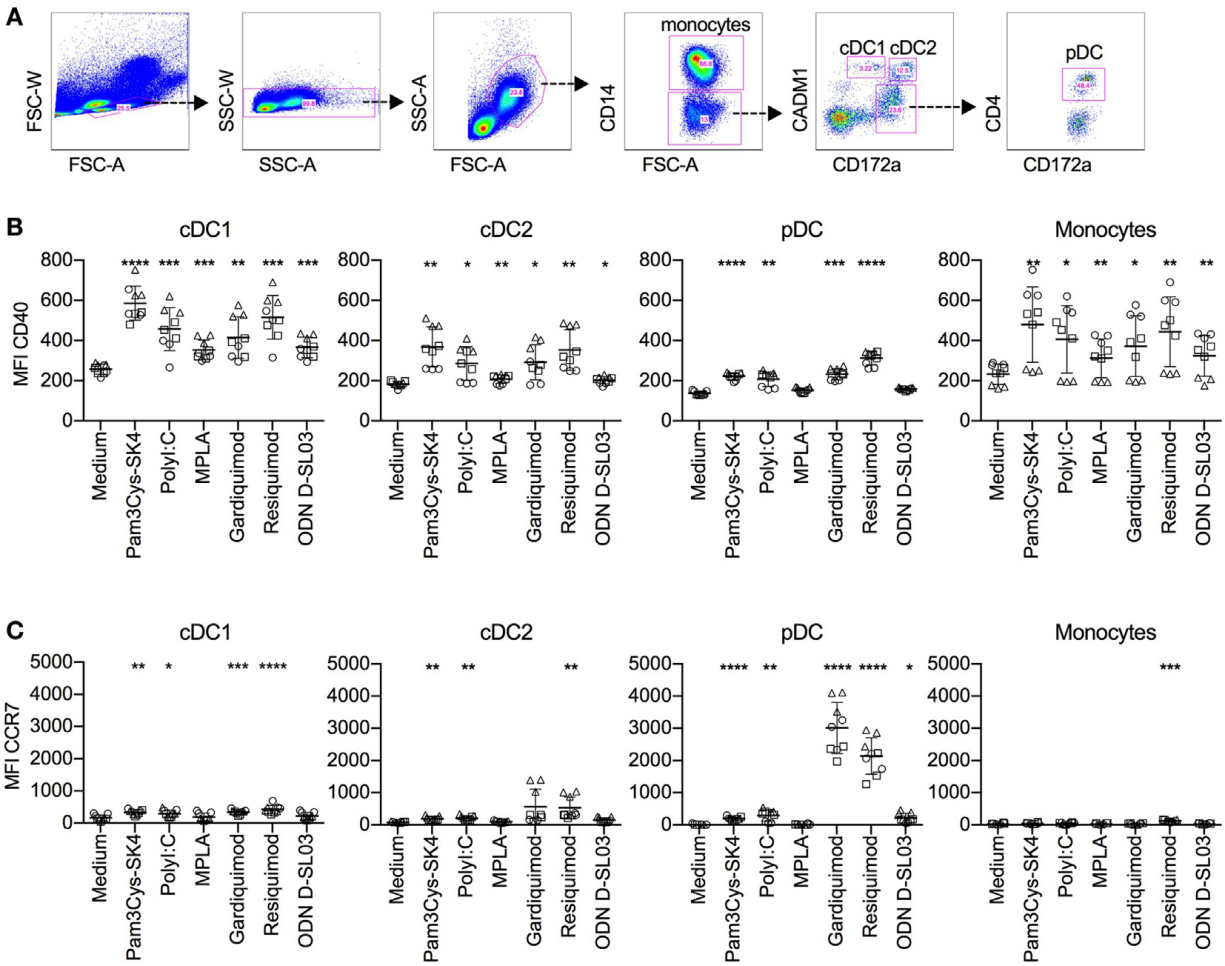
Activation marker response of DC subsets after stimulation with toll-like receptor (TLR) ligands. Peripheral blood mononuclear cells were cultured with TLR ligands for 5 h, and activation marker expression on mononuclear subsets was determined using five-color flow cytometry. **(A)** Gating strategy for identifying conventional DC1 (cDC1), cDC2, and plasmacytoid dendritic cell (pDC) subsets. After doublet exclusion and forward/side scatter gating on large cells, monocytes were identified as CD14^+^ cells, cDC1 as CD14^−^CD172a^low^CADM1^+^, cDC2 as CD14^−^CD172a^high^CADM^+^, and pDC as CD14^−^CD172^+^CD4^+^. **(B)** CD40 levels. **(C)** CCR7 levels on mononuclear cell subsets. The figure shows biological replicates of three independent experiments. Statistical significance was calculated using ANOVA followed by Tukey’s test (****P < 0.0001; ***P < 0.001; **P < 0.01; *P < 0.05).

In the perspective of vaccine development, and to compare B cell TLR responsiveness to other antigen-presenting cells, we decided to investigate activation markers of blood dendritic cell subsets and monocytes after stimulation with the TLR ligands used for the B cell stimulations. The subsets definitions are shown in Figure 7A and are based on previous work (26, 29). After 5 h of stimulation with all ligands tested, CD40 expression was increased on cDC1 cells and monocytes. cDC2 cells responded to all ligands, with the exception of CpG ODN D-SL03. CD40 was also upregulated on pDCs in response to most ligands, with the exception of MPLA. The highest responses were seen with resiquimod (Figure 7B). CCR7 was not induced in cDC1 cells by MPLA and ODN D-SL03. Also, in cDC2 and pDC, MPLA was unable to induce CCR7 (Figure 7C). Interestingly, high induction of CCR7 occurred in pDCs, particularly after stimulation with gardiquimod and resiquimod.

## Discussion

It is well established in mouse models that TLR stimulation of B cells provides important signals for the differentiation of naïve B cells into antibody-producing cells in response to both T-dependent and -independent antigens. In this process, TLR signals promote germinal center formation, affinity maturation, and isotype switching (13, 38–42). Targeting these pathways is relevant to improving vaccines, understanding humoral responses to microbes, treating autoimmunity, and even understanding B cell malignancy development.

We have shown that porcine B cells respond to triggering by several TLR ligands, the strongest being TLR2/X, TLR7/8, and TLR9. These findings are comparable with those for human B cells, although some differences were observed. In human B cells, proliferation induced by TLR7/8 ligands was pDC-dependent (37). With porcine cells, gardiquimod, described as pure TLR7 ligand, also lost its activity when pure B cells were compared with PBMCs, indicating a possible role for pDCs. Resiquimod, described as TLR7/TLR8 ligand, also induced high responses in purified porcine B cells. Pure porcine B cells responded to CpG similarly to their human counterparts. Human B cells are also stimulated *via* TLR3 (14), which did not or very weakly occur in the porcine model. Some studies described B cell activation following stimulation with TLR2/X ligands (14, 43), but our work found that proliferation of porcine cells is higher. Murine B cells responses have been reported to be much stronger following TLR4 as compared to TLR2/X, TLR7, and TLR9 stimulation (12, 44), in contrast with porcine and human B cells. These differences need to be considered when selecting TLR ligands as potential vaccine adjuvants targeting B cells.

It is interesting to note that in all three species, TLR7 and TLR9 expression dominates in B cells, indicating an important role in sensing viral infections. This possibility is supported by *in vivo* studies in mice (20, 22–24).

Compared to whole PBMCs, purified B cells responded in a more homogenous manner. Again, stimulation through TLR2/6, TLR7/8, and TLR9 induced high levels of proliferation, confirming a direct triggering of their receptors on B cells. Of course, this fact does not exclude the possibility that other signals such as IFN-α or IFN-λ derived from pDCs may further enhance B cell responses to such ligands, as described in humans (45).

Looking closer into porcine peripheral B cells, we identified subsets based on expression of IgG and IgM, CD21, and CD11R1. These included a major CD21^+^IgM^+^ subset termed P1, probably representing mostly naïve B cells. Nevertheless, as there is no marker for memory B cells available in the pig, we cannot exclude the presence of some IgM expressing memory B cells. The second subset expressed surface IgG (subset P2) and must therefore have undergone isotype switching indicating memory status. Finally, we identified two novel putative B1-like subsets, both being larger in size, expressing high levels of IgM, CD80/86, and having a higher level of spontaneous PLC-γ2 phosphorylation, characteristics also found with B-1 cells in other species (32–34, 46). Within these B-1 like cells, we found two clearly distinct subsets, a major population of B cells expressing CD11R1 (probably representing the porcine homolog of CD11b) and CD14, reminiscent of a B-1 cell subset also described in human blood (32). Interestingly, the majority of both B-1 like subsets expressed high levels of CD11c, a molecule typically expressed on monocytic cells and DC in the pig (26, 47). However, the P3 and P4 subsets can be classified as B cells based on their expression of IgM and CD79b (data not shown) but lack of CD115 and CD172a. It should be noted that CD5 has been ruled out as a marker for porcine B-1 cells (48), and was therefore not included in our analyses. Clearly, future studies are required to identify their relationship to murine and human B-1 cells, their tissue distribution, B-cell receptor rearrangement, and function.

The data in the present manuscript demonstrated that all porcine B-cell subsets are activated by TLR2 and TLR7 ligands, although some differences were found. Our data indicate that in the pig naïve and memory conventional B cells respond similar to TLR ligand stimulation in terms of CD25, CCR7 upregulation and proliferation. The CD11R1^+^ B1-like subset was the most potent in terms of proliferative responses, relating to its known proliferative capacity (49, 50). This contrasted with the CD11R1^−^ B1-like subset, which was generally the least TLR-responsive subset.

Analysis of sorted B-cell subset surprisingly demonstrated that only the porcine B-1 like subsets P3 and P4, but not the major CD21^+^IgM^+^ P1 subset, were able to secrete IgM following TLR stimulation. This contrasted with data comparing *in vitro* secretion of Ig by murine B cell subsets. While these studies generally showed a higher IgM secretion by B-1 and marginal zone (MZ) B cells, also follicular B cells readily produce all Ig isotypes including IgM following TLR stimulation (12, 50). Nevertheless, comparing B cell subset from different anatomical sources should be interpreted with caution. Also contrasting to human B-1 cells isolated from the peripheral blood (33), we found that neither of the porcine B-1 like subsets constitutively secreted IgM. Nevertheless, the levels of spontaneous IgM secretion were also very low in murine B1 cells (50).

We also analyzed a possible differential expression of TLR transcripts on porcine B cells and found possible differences in the expression of TLR3 and TLR4. While TLR3 transcripts were only found on the B-1 like subsets P3 and P4, TLR4 appeared to be restricted to conventional B cells. The functional consequence of this requires follow-up studies but these data further support the identification of distinct B cell subsets. In murine B cell, TLR expression on different B cell subsets was overall similar with a dominating expression of TLR7 and TLR9 in all subsets (12, 50). Only in MZ, B cells elevated TLR3 transcripts were found (50).

The interpretation of our data on B-1 like cells in the pig requires future studies defining their identity and functional role. It is obvious that species-specific differences in the biology of innate like B cells exist, even when comparing laboratory mice with wild living mice species (51).

As in human B cells, we found that in porcine cells, resiquimod and CpG induced polyclonal and antigen-specific antibody responses in the absence of antigen, demonstrating TLR-driven differentiation of memory B cells into antibody-producing cells. CpG was particularly efficient in this process. Contrary to *in vitro* findings using human B cells (37), depletion of pDCs from the cultures did not abrogate secretion of IgM or FMDV-specific IgG antibodies. On the contrary, we even detected enhanced responses to resiquimod suggesting that pDC produce inhibitory factors. It is unlikely that IFN type I represents such an inhibitory factor as CpG stimulation is by far the most potent IFN-α inducer for porcine pDC (25, 26, 52), and pDC depletion had no influence on CpG-induced antibody responses. On the other hand, the observations that purified B cells poorly responded to TLR stimulation in terms of antibody production indicates that other co-stimulatory factors are important. For restimulation of FMDV-specific memory B cells, we have previously identified T-cell-derived IL-2 and monocyte-derived BAFF to be essential (36). It is possible that such factors are also important for TLR-driven antibody responses.

In the light of previous findings showing the importance of innate activation for potent B cell response, we also evaluated ligands targeting B cells for their ability to activate dendritic cells. We found high responsiveness of cDC1, cDC2, and pDCs in terms of CD40 induction, with the same ligands found to be potent B cell activators. Of particular interest are gardiquimod and resiquimod, as they strongly induced CCR7 expression in pDCs. This would promote their ability to migrate to lymphoid tissue, where IFN-α secreting pDCs could impact B cell differentiation.

We conclude that combinations of such ligands are promising as vaccine adjuvants are able to promote T and B cell responses. While such approaches may be rewarding for pig vaccines, the greater similarity of some aspects of the porcine and human immune systems compared to mice (29, 53–56) is an argument for pigs as an additional animal model for human vaccines or immunotherapeutics.

## Ethics Statement

The animal experiments were performed according to the local law and were approved by the Ethical Committee for Animal Experiments of the Canton of Berne.

## Author Contributions

RB conceived the study, designed and performed experiments, and wrote the manuscript. SP designed and performed experiments. AS conceived the study and wrote the manuscript.

## Conflict of Interest Statement

The authors declare that the research was conducted in the absence of any commercial or financial relationships that could be construed as a potential conflict of interest.
